# Ovarian function and response to gonadotropins after prolonged perfusion of whole ewe ovaries in a bioreactor

**DOI:** 10.1007/s10815-025-03432-6

**Published:** 2025-02-26

**Authors:** Prajakta Hatekar, Panagiotis Tsiartas, Lucía de Miguel Gómez, Claudia Mateoiu, Edina Sehic, Mats Hellström, Pasquale Patrizio, Randa Akouri

**Affiliations:** 1 Laboratory for Transplantation and Regenerative Medicine, Sahlgrenska Academy, University of Gothenburg, Gothenburg, Sweden; 2 Institute of Clinical Sciences, Department of Obstetrics and Gynecology, Sahlgrenska Academy, University of Gothenburg, Gothenburg, Sweden; 3 Karolinska Institute, Department of Clinical Science, Intervention and Technology: Karolinska Institutet, Institutionen För Klinisk Vetenskap, Intervention Och Teknik, Stockholm, Sweden; 4 Stockholm IVF-EUGIN, Stockholm, Sweden; 5 Department of Pathology, Sahlgrenska University Hospital, Gothenburg, Sweden; 6 Health Innovation Labs By Sahlgrenska Science Park, Gothenburg, Sweden; 7 Miller School of Medicine, Division of Reproductive Endocrinology & Infertility, Department of Obstetrics, Gynecology and Reproductive Sciences, University of Miami, Miami, USA; 8 Department of Obstetrics and Gynecology, Sahlgrenska University Hospital, Gothenburg, Sweden

**Keywords:** Ovarian perfusion, Ex vivo culture, Bioreactor, Gene expression, Fertility preservation

## Abstract

**Purpose:**

Fertility preservation for pre-pubertal girls undergoing gonadotoxic cancer treatments and women with systemic cancers at high risk for ovarian metastasis remains limited. Current options, such as ovarian cortex transplantation, risk reintroducing malignant cells. This study presents a novel approach focusing on ex vivo folliculogenesis and mature oocyte retrieval for cryopreservation, mitigating this risk.

**Methods:**

This experimental study optimized an ex vivo ovarian perfusion system in sheep, refining gonadotropin stimulation to yield mature oocytes. Eleven ovaries were divided into two experimental subgroups: Group 1 (*n* = 5) and Group 2 (*n* = 6). Both groups were perfused in a bioreactor for 4 to 8 days under distinct perfusion protocols, differing in gonadotropin administration overnight—Group 1 did not receive overnight gonadotropin stimulation, whereas Group 2 received basal gonadotropin stimulation overnight. Assessments included follicular proliferation, oocyte maturity, apoptosis, ovarian function-related gene expression, and the levels of hormones, metabolites, and electrolytes in the culture medium, compared across subgroups.

**Results:**

The protocol without overnight ovarian stimulation yielded mature MII oocytes, despite fewer secondary follicles and overexpression of the pro-apoptotic *BAX* gene. Conversely, ovaries with overnight stimulation yielded mostly GV-MI oocytes and exhibited reduced secondary follicle proliferation and higher *HIF1A* expression. Hormone levels, metabolites, and electrolytes remained stable across groups and time.

**Conclusions:**

This study is the first to report the successful harvesting of MII oocytes following extended ex vivo perfusion of intact ewe ovaries, highlighting the potential of the perfusion model to support advanced follicular development. Further investigations are warranted to elucidate underlying mechanisms and refine protocol efficiency.

**Supplementary Information:**

The online version contains supplementary material available at 10.1007/s10815-025-03432-6.

## Introduction

Improved cancer therapies have led to better survival rates following diagnosis. However, common side effects include impaired reproductive health, particularly for women [1]. These unintended consequences have become a significant concern for cancer survivors, and infertility in particular is a significant disruptor of quality of life after cancer, resulting in profound psychological and emotional distress [1, 2]. Over the years, various fertility preservation strategies have successfully been developed for women requiring gonadotoxic cancer treatment, including ovarian transposition, ovarian suppression with gonadotropin-releasing hormone (GnRH) analogues, oocyte and/or embryo vitrification, and ovarian cortex transplantation (OCT) [3–5]. However, oocyte and/or embryo vitrification, the most commonly adopted options, require gonadotropin stimulation and are therefore not suitable for pre-pubertal girls who have not yet reached sexual maturity [4]. OCT is currently the only available fertility preservation option for pre-pubertal girls and for women who require immediate cancer treatment [4, 6]. Despite its potential, OCT poses significant risks, particularly for patients with systemic hematological malignancies, as cryopreserved autologous ovarian tissue may harbor malignant cancer cells, increasing the risk of reintroducing malignancy upon transplantation [7–9].

For these reasons, new fertility preservation methods for hematologic cancer patients and for pre-pubertal girls are warranted. Despite advancements of in vitro maturation protocols to recapitulate follicle development across various animal models, including domestic animals, primates, and humans, achieving complete folliculogenesis resulting in mature oocyte production and ultimately into a viable offspring remains limited to mice [10, 11]. Challenges arise in larger species due to the prolonged and sizable follicle development, necessitating extended culture periods [12–14]. In this context, organ perfusion using bioreactor technology under normothermic conditions offers a promising alternative. Unlike traditional methods, bioreactors enable the precise control of environmental factors such as oxygenation, temperature, and nutrient supply, replicating physiological conditions for prolonged ovarian function ex vivo [15–17]. This approach opens the possibility of explanting an ovary from a cancer patient, perfusing it with gonadotropins in a bioreactor, and supporting follicular development, oocyte maturation, and subsequent oocyte retrieval ex vivo. Although this method is still in its early stages of development, preclinical trials with sheep ovaries have been encouraging by showing sustained follicle viability within the ex vivo perfused ovaries and the ability to retrieve immature oocytes following the normothermic perfusion of up to 7 days employing two distinct gonadotropin stimulation protocols [15]. Ex vivo folliculogenesis using a bioreactor was initially described by our group [17], with preliminary findings on sheep ovaries perfused for up to 7 days. In this study, we refined the protocol for ovarian stimulation, extending the stimulation period to 8 days with the goal of obtaining mature (metaphase II) oocytes. Bioreactor perfusion overcomes the diffusion limitations, hypoxic stress, and static hormonal environments of in vitro maturation (IVM) and organ culture systems, offering a dynamic and physiologically relevant platform that supports prolonged follicular development and oocyte maturation in larger species. The potential for bioreactor technology to overcome the limitations of IVM while mitigating the risks associated with OCT highlights its novelty and transformative potential in fertility preservation.

To further optimize this novel procedure, we designed the current study with two main objectives: First, we aimed to refine the gonadotropin stimulation protocol by optimizing dosage and administration intervals to better mimic in vivo gonadotropin secretion, thereby improving the retrieval of mature oocytes. Second, we sought to comprehensively assess follicular integrity and oocyte maturity, including analysis of targeted gene expression markers after perfusion. Specifically, we focused on FSH receptor (*FSHR*), LH/choriogonadotropin receptor (*LHCGR*), anti-Müllerian hormone (*AMH*), and insulin-like growth factor 1 (*IGF-1*), which are regulators of follicular development, ovarian function, and cell growth. Additionally, we examined B cell lymphoma protein 2 (*BCL-2*) and Bcl-2 associated X protein (*BAX*), which are known mediators of apoptosis, as well as hypoxia-inducible factor 1 subunit alpha (*HIF1A*), a pivotal factor in cellular hypoxic responses. Finally, superoxide dismutase 1 (*SOD1*) and tumor necrosis factor alpha (*TNFA*) were analyzed due to their roles in endothelial cell-related pro-inflammatory and antioxidant pathways.

Moreover, the study considered the potential occurrence of negative cold ischemic events during the interval between ovarian explantation and reperfusion. Negative cold ischemic events refer to the detrimental effects on tissues or organs caused by the lack of oxygen and nutrients during cold storage. These collective efforts aim to construct a study framework that will facilitate ex vivo perfusion and gonadotropin stimulation investigations relevant to human subjects. An effective ex vivo approach for obtaining mature oocytes holds significant promise for fertility preservation in pre-pubertal girls and for young women with systemic cancer who face challenges in delaying cancer treatment or relying exclusively on OCT.

## Materials and methods

### Ovary isolation and back table perfusion

A total of 22 ovaries were obtained from 11 sexually mature ewes (8–12 months old) of mixed breeds at a local slaughterhouse between October 2021 and November 2022. Immediately after euthanasia, 5-mL blood samples were collected from each animal and kept on ice during transportation to the laboratory. Blood samples were then centrifuged at 1500 g for 20 min, and plasma was stored at − 20 °C for further analysis.

To minimize contamination, all ovaries with intact vascular pedicles were rinsed once at the slaughterhouse, by dipping in a chlorhexidine solution dissolved in normal saline (0.001%; Fresenius Kabi, Uppsala, Sweden), followed by three dips in a sterile saline solution (0.9%; Braun, Melsungen AB, Germany).

**Fig. 1:**
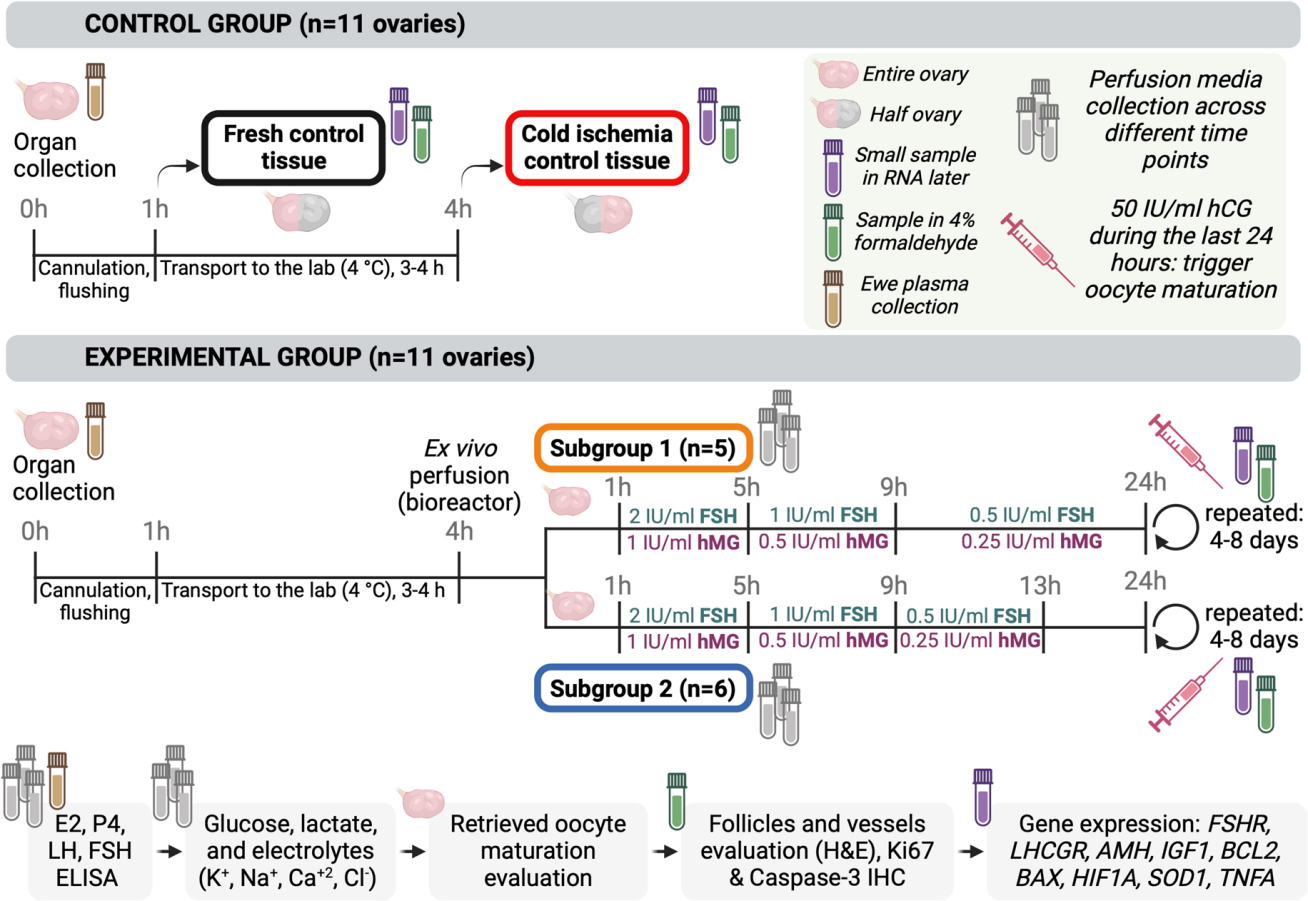
Study design. Twenty-two ovaries were obtained from sexually mature ewes and divided into two main groups: control (*n* = 11) and experimental (*n* = 11). All ovaries underwent cannulation and flushing at the collection site before transportation to the laboratory within a 3- to 4-h timeframe. In the control group, half of each ovary served as fresh control tissue, while the other half served as control tissue to assess potential cold ischemia damage that occurs during transportation to the laboratory. In the experimental group, all ovaries were connected to a bioreactor for ex vivo perfusion. These ovaries were further divided into two experimental subgroups, each undergoing distinct perfusion protocols. Both protocols (established for 24 h and then repeated until experiment termination) involved hormonal stimulation with follicle-stimulating hormone (FSH) and human menopausal gonadotropin (hMG), with concentration halved every 4 h (starting concentration 2 IU/mL FSH and 1 IU/mL hMG). However, after 12 h, only subgroup 2 maintained FSH/hMG stimulation overnight. hCG, human chorionic gonadotropin. Figure created with Biorender

The collected ovaries were divided into an experimental group (*n* = 11) and a control group (*n* = 11) (Fig. 1). The ovarian artery was separated from the ovarian vein using a dissection microscope (Nikon, Tokyo, Japan), and all ovaries were initially perfused at the slaughterhouse with 2 mL of cold (4 °C) sterile phosphate-buffered saline (PBS, Gibco, London, UK) supplemented with heparin (50 IU/mL; Apoteket AB, Stockholm, Sweden), xylocaine (0.04 mg/mL; Astra Zeneca, Gothenburg, Sweden), piperacillin/tazobactam (10 µg/mL and 1.25 µg/mL; Stragen Nordic, Hillerød, Denmark), and Gibco’s antibiotic–antimycotic solution (1%; Thermo Fisher Scientific, Stockholm, Sweden) until the organ blanched and clear liquid was observed flowing through the ovarian vein. The venous outflow was collected and stored at − 20 °C for further analysis. For the ovaries included in the experimental group, the ovarian artery was then cannulated (24 G; Becton Dickinson Infusion Therapy AB, Helsingborg, Sweden) using a dissection microscope (Nikon, Tokyo, Japan). The cannula was then fixed using a sterile 4–0 silk suture while the venous outflow was left open. Ovaries allocated to the experimental group were placed in a cold (4 °C) sterile PBS solution and transported on ice to the laboratory. Ovaries in the control group after the flushing were immediately dissected, and a small piece (approximately 2 mm^3^) of cortex tissue was placed in RNAlater (Qiagen, Hilden, Germany) for subsequent gene expression analysis, while a larger piece (approximately 4–5 cm^3^) was placed in 4% formaldehyde serving as fresh control tissue for histomorphological and immunohistochemistry examination. The remaining half of each control ovary was transported to the laboratory with the same conditions used for the ovaries in the experimental group. At the laboratory, the remaining half of each control ovary was further divided into two pieces; a small cortex tissue piece was placed in RNAlater (Qiagen), while the other piece was placed in 4% formaldehyde [15].

The average time between euthanasia, ovarian artery isolation, cannulation, and vascular perfusion was between 30 and 60 min, while the average cold ischemia time during the transport to the laboratory ranged from 180 to 240 min.

### Implementation of a modified closed circulation perfusion system for ex vivo ovarian perfusion

**Fig. 2:**
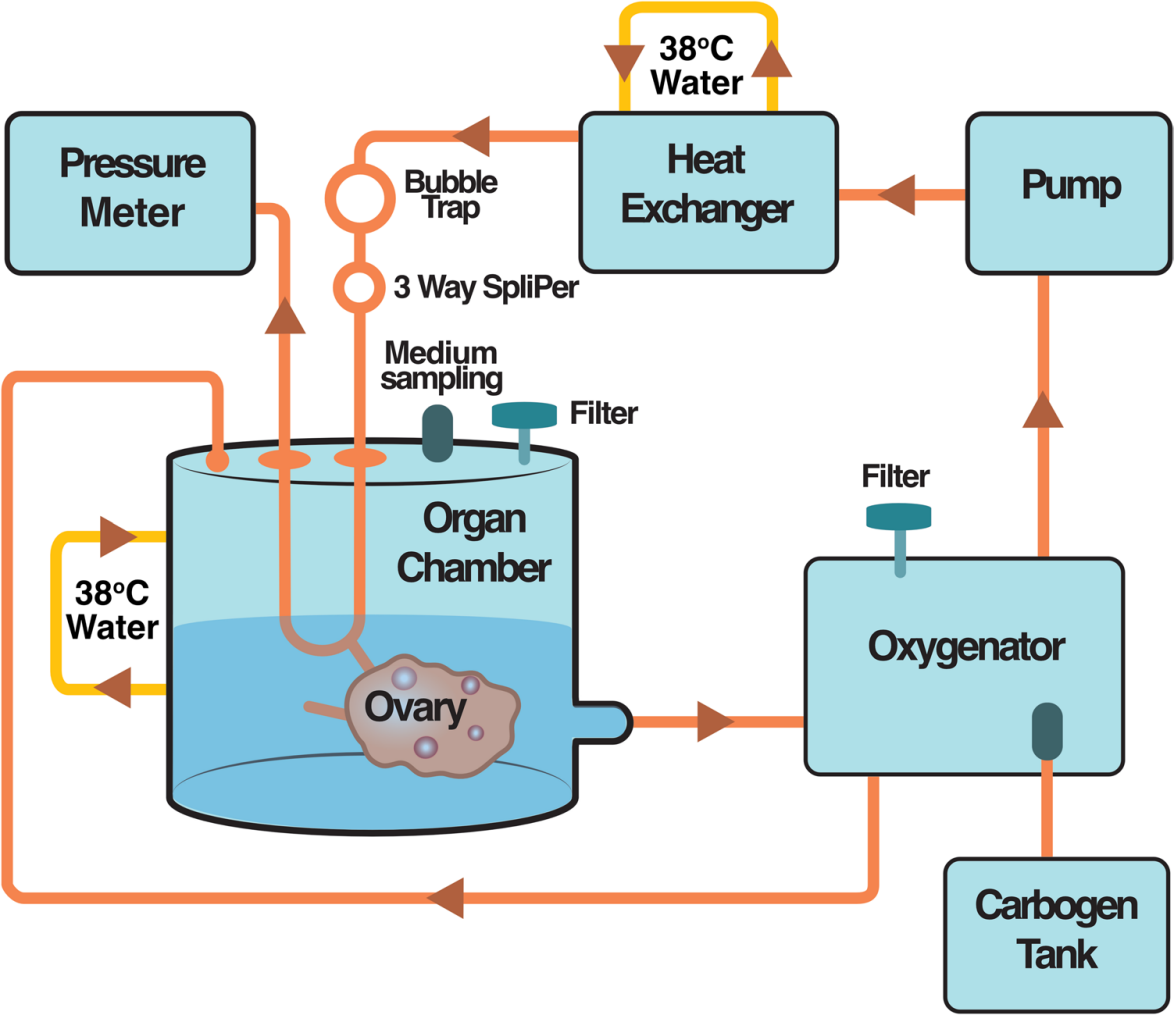
Schematic representation of a bioreactor system designed for organ perfusion. The setup includes a peristaltic pump for controlled fluid circulation, an oxygenator for maintaining oxygen levels in the perfusion medium, and a heat exchanger for regulating temperature. The organ perfusion chamber houses the perfused ovary, which is completely submerged within the perfusion medium. This system enables precise control of environmental conditions to support the viability and function of the perfused organ [15]

The ovaries assigned to the experimental group underwent ex vivo perfusion in a modified closed circulation bioreactor utilized in our previous study [15]. The perfusion system involved keeping the cannulated ovary in the media reservoir, fully immersed in the perfusion medium as depicted in Fig. 2 (Fig. 2). Thus, the media reservoir also acted as the organ chamber. The bioreactor system enabled recirculation of the perfusate (closed system), ensuring a sterile environment with adjustable monitoring of temperature, oxygenation, perfusion pressure, and flow rate throughout the experiment. The bioreactor comprised of three distinct circuit loops: (1) the organ chamber, which was integrated with the media reservoir; (2) Carbogen 5 gas (gas mixture of 95% O_2_, 5% CO_2_; Linde, Solna, Sweden), which provided buffering but also contributed to oxygenation of the perfusion media; and (3) warm circulating water (38 °C) in a jacketed system that maintained a constant physiological temperature for both the perfusate and the organ throughout the experiment. A peristaltic pump facilitated the circulation of the perfusate through the oxygenator, including a bubble trap, and directed it to the ovary in the organ chamber. To prevent potential damage to the ovarian vasculature, the perfusion pressure was kept below 80 mmHg by adjusting the flow rate to 1.53 mL per min during the entire perfusion period [15].

### Experimental design and perfusion protocols for ovarian stimulation

The ovaries in the experimental group were divided into two subgroups, each receiving a distinct gonadotropin stimulation protocol. Based on the daily peaks of gonadotropin secretion in cycling ewes [16] and the findings from our previous study [15], both experimental subgroups received different daily doses of follicle-stimulating hormone (FSH) and human menopausal gonadotropin (hMG) media supplementation as detailed in the subsequent description. The difference between subgroups was that subgroup 1 received continuous gonadotropins including overnight administration while subgroup 2 had an overnight pause (Fig. 1).

Ovaries in subgroup 1 (*n* = 5) were perfused for 4 to 8 days with a medium composed of M199 (Thermo Fisher Scientific) supplemented with 2% bovine serum albumin (Roche Diagnostic GmbH, Mannheim, Germany), piperacillin/tazobactam (10 µg/mL and 1.25 µg/mL; Stragen Nordic), Gibco antibiotic–antimycotic solution (Thermo Fisher Scientific), 12 mL/L sodium bicarbonate (7.5%; Thermo Fisher Scientific), 10% fetal bovine serum (FBS) (Thermo Fisher Scientific), IGF-1 (50 ng/mL, Peprotech, Stockholm, Sweden), and HEPES (Sigma Aldrich, Stockholm, Sweden). After an initial perfusion of 1 h without gonadotropins, FSH (Gonal-F, Merck AB, Solna, Sweden) and hMG (Menopur, Ferring Pharmaceuticals, Malmö, Sweden) were added in the medium in a single dose of 2 + 1 IU/mL, respectively, for 4 h, followed by 1 + 0.5 IU/mL for the next 4 h and 0.5 + 0.25 IU/mL for an additional 15 h. This cycle was repeated every 24 h until the end of the perfusion experiments.

Ovaries in subgroup 2 (*n* = 6) were perfused for 4 to 8 days with the same medium and protocol used for subgroup 1, except for not receiving the 15-h low-dose gonadotropin stimulation that was replaced by a 4-h perfusion with low-dose of gonadotropins (0.5 + 0.25 IU/mL of FSH and hMG respectively) followed by 11 h overnight pause of hormonal exposure. These conditions were repeated every 24 h during the perfusion experiments.

To trigger final oocyte maturation in both experimental subgroups, human chorionic gonadotropin (hCG) 50 IU/mL (Pregnyl, MSD Sverige AB, Stockholm, Sweden or Ovitrelle, Merck AB, Solna, Sweden) was added to the medium during the last 24 h of perfusion before ovum pick-up (OPU). OPU was performed by retrieving newly visible follicles with a diameter between 3 and 5 mm through gentle manual aspiration using an 18-G needle connected to a tube with an inner diameter of 2 mm [17].

During the experiments, 10 mL of the perfusion medium was sampled from the organ chamber prior to medium changes at ten different time points: 20 min, 1 h, 5 h, 9 h, 24 h, 36 h, 48 h, 60 h, 72 h, and 80 h, until the end of perfusion (prior to any medium change) and immediately frozen at − 20 °C for further biochemical analysis, as described below.

At the end of the ex vivo perfusion, a small ovarian piece of cortical tissue, about 2 mm^3^, was directly placed in RNAlater for future gene expression analysis, while the remaining ovarian tissue was fixed in 4% formaldehyde for later histopathological analysis.

### Assessment of follicular steroidogenic activity and reproductive cycle phase in ewes

To assess the follicular steroidogenic activity and reproductive cycle phase of each ewe, concentrations of hormones were measured in the undiluted sampled media, plasma, and liquid collected after gently flushing the organ at the slaughterhouse. The following ELISA kits, specific for sheep, from Reagent Genie (Dublin, Ireland) were used: estrogen (#SHFI00063; sensitivity, 9.375 pg/mL; detection range, 15.625–1000 pg/mL), progesterone (#SHFI00062; 18.75 pg/mL, 31.25–2000 pg/mL), FSH (#SHFI00026; 1.875 mIU/mL, 3.125–200 mIU/mL), and luteinizing hormone (LH; #SHFI00032; 0.563 mIU/mL, 0.938–60 mIU/mL).

### Measurement of ovarian metabolic activity

To assess ovarian metabolic activity, concentrations of glucose, lactate, potassium, sodium, calcium, and chloride were measured in the perfusion medium using a standard blood-gas analysis using the RapidPoint 500 Blood Gas System (Siemens Healthcare, Erlangen, Germany), following the manufacturer’s protocols.

### Evaluation of oocyte maturation stages

Retrieved oocytes were immediately examined by two experienced embryologists, who were blinded to the experimental subgroups. The oocytes were classified based on their maturation stages into germinal vesicle (GV), metaphase I (MI), and metaphase II (MII) oocytes. Oocyte maturity assessment was performed using a stereomicroscope. The number of retrieved oocytes, recovery rates, and their distribution across these maturation stages were meticulously recorded.

### Assessment of ovarian tissue integrity through histomorphology and immunohistochemistry

Formaldehyde-fixed ovarian samples included fresh control tissue (collected and fixed at the slaughterhouse), cold ischemia control tissue (collected and fixed after transport to the laboratory), and the experimental ex vivo perfused ovaries. These biopsies underwent dehydration and paraffin embedding followed by 4 µm sectioning and stained with hematoxylin and eosin (H&E) as per standard protocols. Each specimen was examined by an experienced pathologist blinded to the sample groups. To prevent duplicate follicle counting, a minimum of ten sections from each ovary were reviewed, with at least a 50-µm separation between each section. Digital scanning of all slides was performed using a NanoZoomer S210 scanner (Hamamatsu Photonics, Hamamatsu, Japan), and the surface area of the ovarian cortex in each section was measured using NanoZoomer Digital Pathology view 2 analysis software (Hamamatsu Photonics). The degree of injury was assessed digitally, based on established pathology parameters [18, 19]. Non-damaged primordial follicles were identified as those with a spherical oocyte and a non-pyknotic nucleus, surrounded by one layer of flattened granulosa cells. Non-damaged primary follicles were small, spherical follicles containing a GV stage oocyte encircled by one to two layers of cuboidal granulosa cells. Secondary non-damaged follicles were larger with several layers of granulosa cells, sometimes having small spaces between them, and a GV stage oocyte. Non-damaged antral follicles were recognized by their fluid-filled antral cavity, with the oocyte situated at the edge in a mound of granulosa cells (cumulus oophorus). Damaged follicles were categorized into grade 1 (G1) when theca and granulosa cells were separated from the follicular edge, showing disruptions and apparent loss of granulosa cells while the oocyte maintained its spherical shape. Grade 2 follicles (G2) exhibited more severe disruptions, loss of granulosa cells, theca cell detachment from the follicle edge, pyknotic nuclei in granulosa cells, and misshapen oocytes, with or without vacuolation or pyknotic nuclei. The overall ovarian histomorphological architecture was evaluated for abnormalities in arteries and veins, such as endothelial detachment, internal elastic membrane rupture, or smooth muscle cell bloating [20, 21].

To study follicular cell proliferation, 3,3ʹ-diaminobenzidine (DAB) immunohistochemistry for Ki-67 (#15,580, 1:100, Abcam, Cambridge, UK) was performed. Apoptosis was confirmed by immunostaining for cleaved caspase 3 (#9661, 1:300, Cell Signaling Technology, BioNordika, Stockholm, Sweden). Antigen retrieval was conducted in a pressure cooker using Diva Decloaker (Biocare Medical, HistoLab, Gothenburg, Sweden) for Ki-67 and citric acid buffer (pH = 6.0) for cleaved caspase 3. Immunohistochemistry analysis was performed using the Mach 3 and Vulcan fast red kits (Biocare Medical) after overnight incubation with the respective primary antibodies. Finally, all slides were cover-slipped with Pertex (Histolab) and scanned using a microscope slide scanner. Proliferation and apoptotic indexes were determined by calculating the percentage of Ki-67- and cleaved caspase 3-positive cells in hotspot areas of the ovarian cortex, considering each follicular subclass separately.

### Quantitative gene expression analysis using digital droplet PCR

For gene expression analysis, we adhered to the Minimum Information for Publication of Quantitative Experiments (MIQE) guidelines for digital droplet PCR [22]. Biopsies kept in RNAlater were weighed, segmented into 30-mg pieces, and homogenized. Total RNA extraction was performed using the RNeasy micro-Kit (Cat No. #74,034; Qiagen). Reverse transcription was performed using the iScript cDNA Synthesis Kit (Cat No. #170–8891; BioRad, Stockholm, Sweden). The PCR mixtures were prepared with EvaGreen primer mix (Cat No. #164–4033; BioRad) and converted into droplets following the manufacturer’s instructions using QX200™ droplet generation oil for EvaGreen (Cat No. #1,864,006; BioRad) and the QX200 droplet generator (BioRad). During partitioning, each reaction was divided into 16,000–21,000 droplets, followed by PCR amplification within a C1000 thermal cycler (BioRad). Data analysis involved measuring the fluorescence intensity of each droplet using a droplet reader and employing Quantasoft software (BioRad) for comprehensive analysis. Negative and positive droplet populations were identified, and the number of copies per microliter was standardized to the reference gene peptidyl‐prolyl cis‐trans isomerase H (*PPIH*). Subsequently, the relative expression levels were determined. Additionally, the primers used in this study were designed and synthesized by Integrated DNA Technologies, with specific sequences detailed in Supplementary Table 1. In this study, we analyzed a set of genes, including *FSHR*, *LHCGR*, *AMH*, *IGF-1*, *BCL-2*, *BAX*, *HIF1A*, SOD*1*, and *TNFA*, focusing on their roles in follicular development, ovarian function, cell growth, apoptosis, hypoxia response, and endothelial cell-related pro-inflammatory and antioxidant processes.

### Statistical analysis

Gene expression analyses entailed group comparisons, utilizing log-transformed outcome values to enhance statistical robustness. The non-parametric Kruskal–Wallis test was selected for these comparisons, with adjustments for multiple comparisons based on Dunn’s criterion. This consistent approach extended to the assessment of histomorphological and immunohistochemistry differences across follicle categories, specifically targeting non-damaged, G1, and G2 follicles. The categorization of blood vessel characteristics into normal or injured states prompted an investigation into their associations with experimental groups, control ovary, and cold ischemia by applying Fisher’s exact test. *P*-values between groups were determined through logistic regression analysis. In exploring changes during ovarian perfusion, mixed models were deployed to analyze observed values of metabolic and electrolytic biomarkers, along with steroid hormones. This approach facilitated the examination of alterations within each experimental subgroup as well as between them across perfusion periods. The reported *P*-values underwent adjustment using Tukey’s criterion to account for multiple comparisons, ensuring the robustness of the statistical findings. All statistical analyses were conducted using GraphPad Prism v. 9.0 (CA, USA), with a predetermined significance level of 0.05 for all tests.

### Ethical approval

Ethical approval for the study was not required, as the ovaries were obtained from ewes bred for food production with the possibility to use discarded organs for research purposes, aligning with the Swedish and European Union ethical regulations.

## Results

### Impact of ovarian perfusion on hormone production

**Fig. 3:**
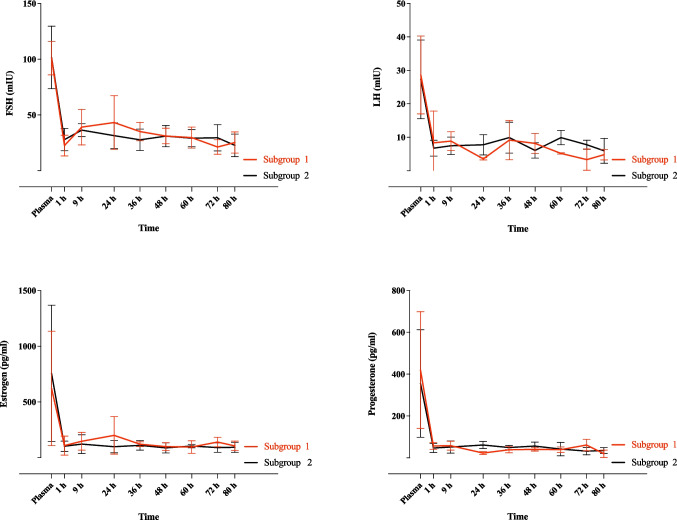
Temporal patterns of gonadotropins (FSH, LH) and steroid hormones (estrogen, progesterone) changes in the perfusion medium at various time points during ovarian perfusion in experimental subgroups 1 and 2. Additionally, plasma concentrations of the hormones before euthanasia of the ewes are illustrated

There were no significant changes in concentrations of estrogen, progesterone, FSH, and LH in both subgroups during the perfusion period. Additionally, both subgroups exhibited comparable temporal patterns of steroid changes throughout the perfusion. Notably, hormone concentrations in the perfusion medium were significantly lower compared to ewe plasma levels (Fig. 3).

### Impact of ovarian perfusion on metabolic and electrolyte biomarkers

**Fig. 4:**
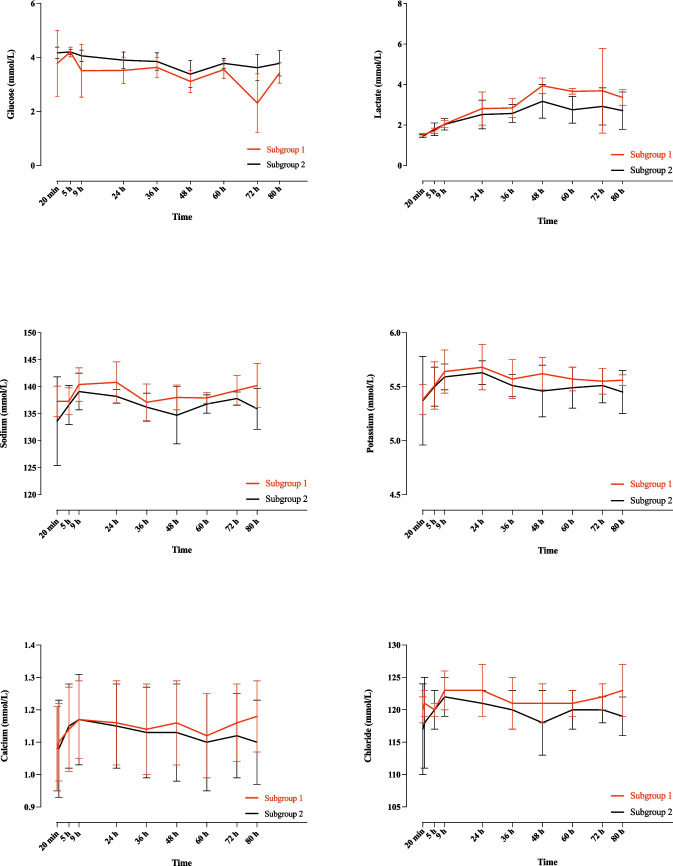
Temporal patterns of metabolic and electrolytic changes in the perfusion medium assessed at different time points during the ovarian perfusion in experimental subgroups 1 and 2

After assessing the perfusion medium through blood-gas analysis, no significant changes were observed in the concentrations of metabolic and electrolyte biomarkers during the perfusion, independent of gonadotropin stimulation protocol used. Both subgroups demonstrated comparable temporal metabolic and electrolytic change patterns throughout the perfusion process (Fig. 4).

### Comparing the impact of cold ischemia and reperfusion: histomorphological changes, proliferation, and apoptosis

**Fig. 5:**
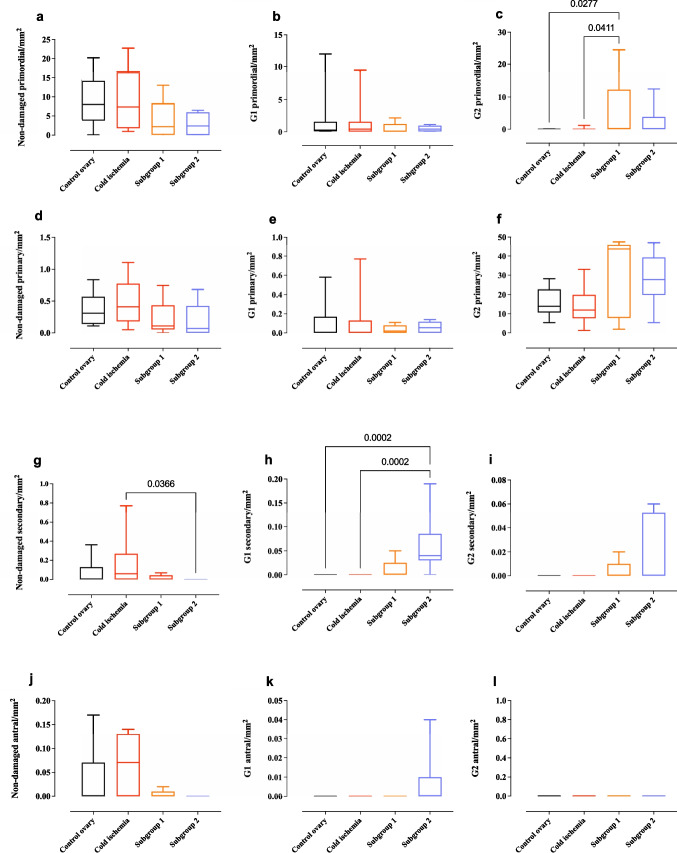
Comparison of ovarian cortex follicular populations among control ovaries, ovaries exposed to cold ischemia, and ovaries subjected to normothermic perfusion. G1, damaged, grade 1 follicles; G2, damaged, grade 2 follicles)

The ovarian cortex follicular population among both subgroups remained preserved without a significant reduction in the number of non-damaged primordial, primary, and antral follicles per square millimeter (Fig. 5a, d, j). However, when compared to cold ischemia samples, the number of non-damaged secondary follicles was significantly lower in the ovarian cortex tissue from experimental subgroup 2 [median (IQR); 0 (0) vs. 0.06 (0.27); *p* = 0.0366] (Fig. 5g). Furthermore, a significantly higher number of damaged, G1 secondary follicles were observed in subgroup 2 compared to both the pre-transportation fresh control ovary [median (IQR); 0.04 (0.01) vs. 0 (0); *p* = 0.0002] and the ovary after cold ischemia [median (IQR); 0.04 (0.01) vs. 0 (0); *p* = 0.0002] (Fig. 5h). Additionally, a significant increase was noted in the number of damaged, G2 primordial follicles per square millimeter after perfusion in subgroup 1 compared to the control ovary [median (IQR); 0.15 (0.07) vs. 0 (0); *p* = 0.027] and cold ischemia [median (IQR); 0.15 (0.07) vs. 0 (0); *p* = 0.041] (Fig. 5c).

The analysis of vessel integrity indicated a significantly higher percentage of injured medullary arteries (66.7% vs. 0%, *p* = 0.001) and veins (50% vs. 0%, *p* = 0.008) only in subgroup 2 compared to the control ovary and the ovary after cold ischemia. However, despite this marked vascular injury within the medullary region, the overall histomorphology of ovarian arteries and veins remained intact across all groups, indicating that the main structural integrity of these vessels was preserved regardless of ischemic conditions or subgroup classification. This suggests that while medullary vessels are more vulnerable to injury in certain conditions, the global ovarian vascular architecture is more resistant to disruption.

Compared with the control ovary, viable primary follicles in subgroup 2 showed significantly lower Ki-67 staining (proliferation marker) [median (IQR); 0 (0) vs. 0.01 (0.05); *p* = 0.008]. Similarly, compared to the ovary after cold ischemia, the viable secondary follicles in subgroup 1 had a significantly lower Ki-67 staining [median (IQR); 0 (0.01) vs. 0.20 (0.7); *p* = 0.035], indicating reduced cell proliferation in these follicular types. Additionally, a significantly higher cleaved caspase 3 staining (apoptosis marker) was observed in damaged secondary follicles in subgroup 2 compared with the control ovary and the ovary exposed to cold ischemia [median (IQR); 0.25 (0.4) vs. 0 (0); *p* = 0.002].

**Fig. 6:**
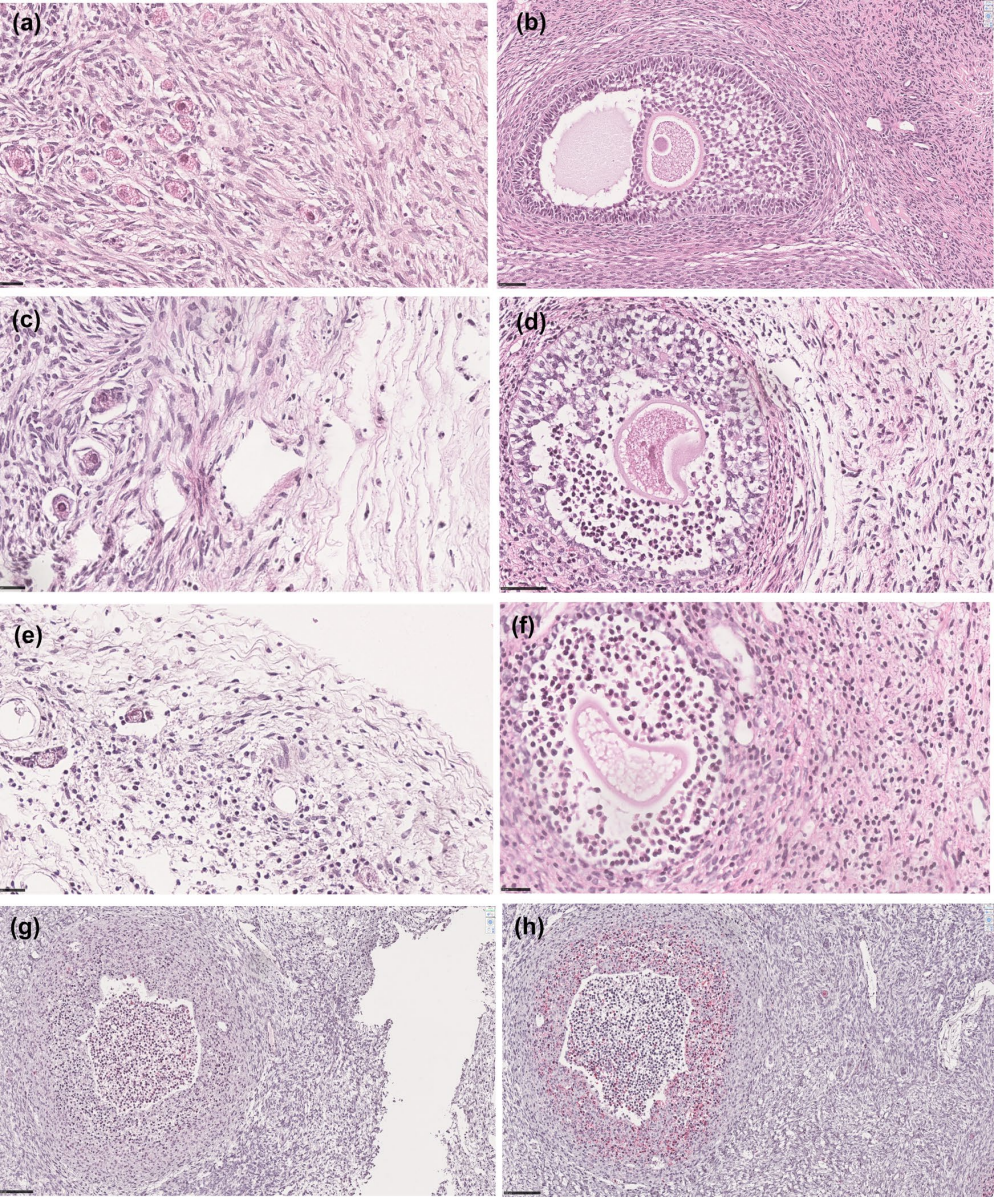
Representative microscopic images of perfused ovaries displaying follicles of various categories with hematoxylin and eosin (H&E) staining. **a** Non-damaged primordial follicles (scale bar = 25 µm); **b** non-damaged antral follicle (scale bar = 50 µm); **c** grade 1 (G1) damaged primordial follicles (scale bar = 25 µm); **d** G1 damaged antral follicle (scale bar = 50 µm); **e** grade 2 (G2) damaged primordial follicles (scale bar = 25 µm); **f** G2 damaged antral follicle (scale bar = 25 µm); **g** follicle with immunohistochemical staining positive for Ki-67 (red) (scale bar = 100 µm); **h** follicle with immunohistochemical staining positive for cleaved caspase-3 (red) (scale bar = 100 µm)

In Fig. 6, histological analysis of perfused ovaries illustrates follicles at different developmental stages, exhibiting varying degrees of damage. Non-damaged primordial and antral follicles appeared intact with well-preserved cellular architecture (Fig. 6a, b). In contrast, G1 and G2 damaged follicles exhibited structural degeneration (Fig. 6c–f). Immunohistochemical staining confirmed proliferative activity in some follicles, as indicated by Ki-67 positivity (Fig. 6g), while cleaved caspase-3 staining highlighted apoptotic cells (Fig. 6h).

### Comparison of oocyte recovery rates and maturation status in the perfused ovaries

**Fig. 7:**
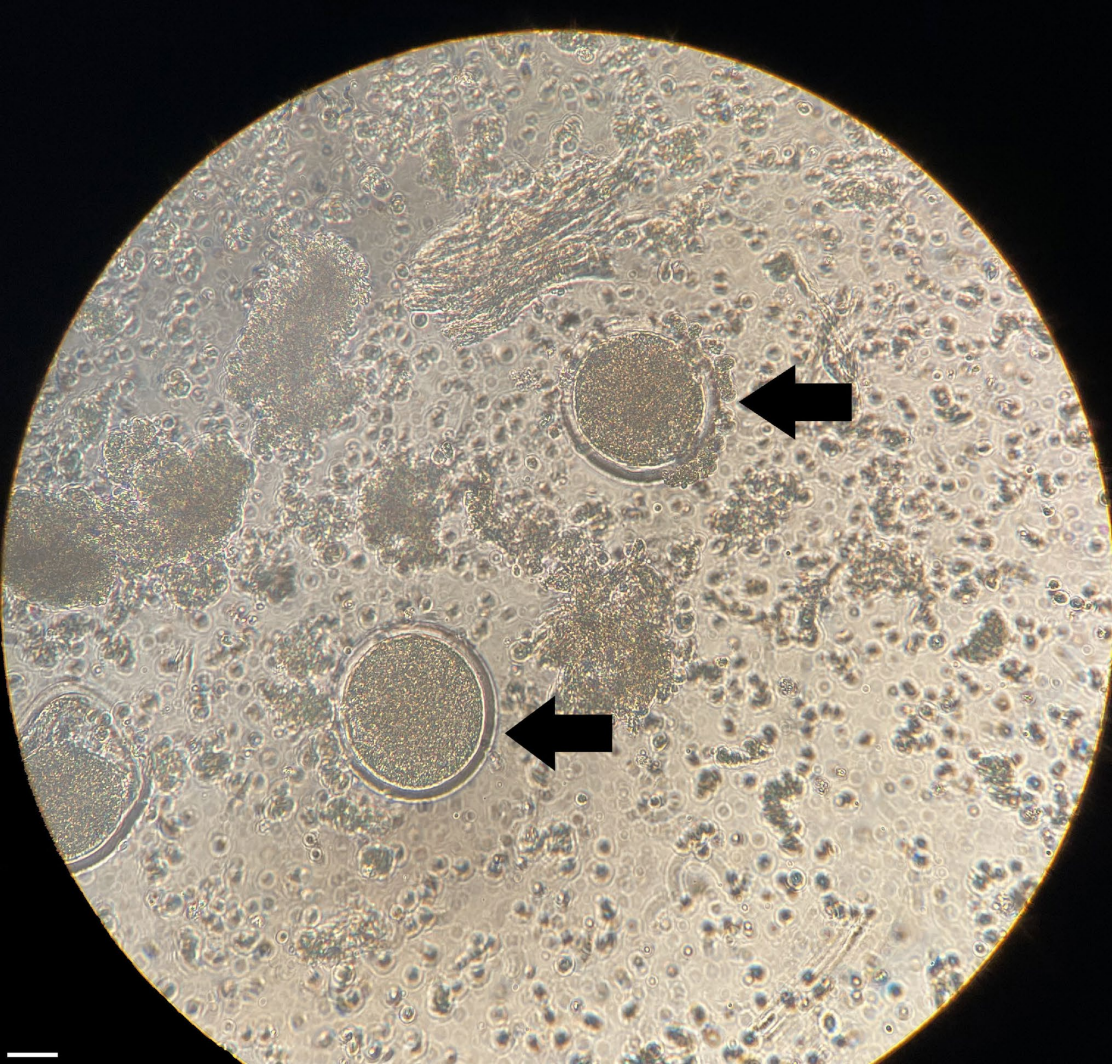
Microscopic image of metaphase II (MII) oocytes (black arrows) captured under bright-field conditions at 200 × magnification. The scale bar represents 50 µm

In experimental subgroup 1, 16 follicles were aspirated, and a total of 8 oocytes were retrieved, resulting in a 50% oocyte recovery rate. Among these, seven oocytes were in stage GV-MI, while one oocyte had degenerated. Conversely, in subgroup 2, 30 aspirated follicles yielded 22 oocytes (73% oocyte recovery rate). Specifically, two of these oocytes were at the MII stage, 19 were at the GV-MI stage, and one was degenerated. Notably, oocytes retrieved from subgroup 2, including those at the MII stage, indicated a more advanced maturation status compared to subgroup 1. Additionally, all retrieved oocytes exhibited homogeneous cytoplasm and were partially covered by cumulus cells (Fig. 7).

### Impact of perfusion on gene expression profiles

**Fig. 8:**
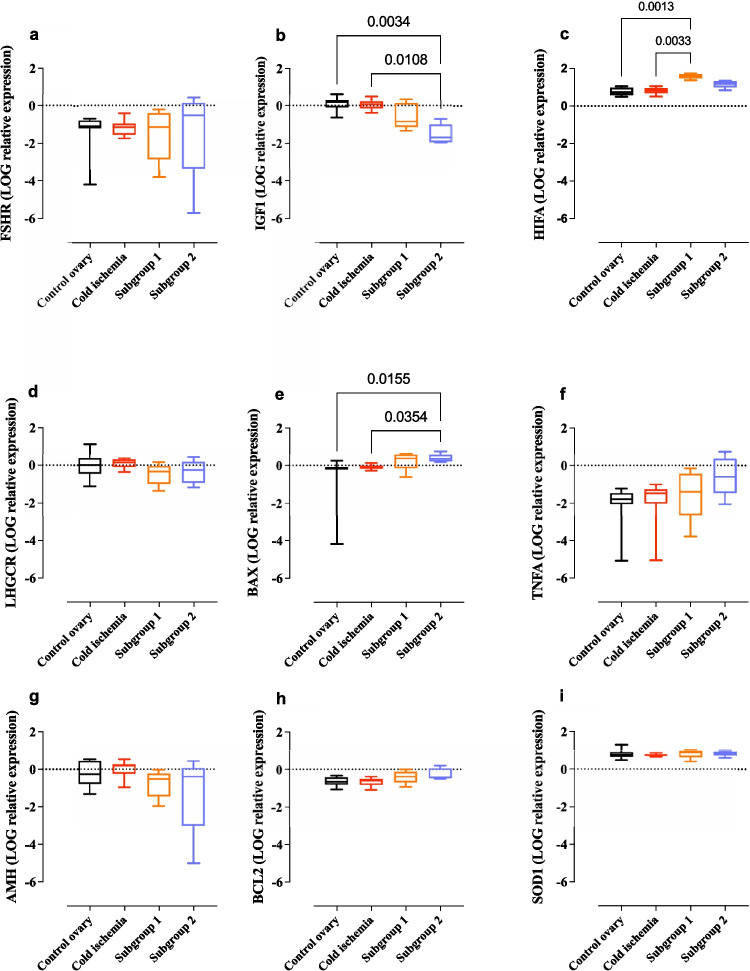
Log-transformed relative mRNA expression values of target genes in ovarian tissue. Gene expression values depict the comparison between experimental subgroups 1 and 2, and their respective paired cold ischemia and control ovarian tissue. Data were analyzed for key genes associated with ovarian function, including **a** follicle-stimulating hormone receptor (FSHR); **b** insulin-like growth factor 1 (IGF1); **c** hypoxia-inducible factor 1 subunit alpha (HIFA); **d** luteinizing hormone/choriogonadotropin receptor (LHCGR); **e** BCL2-associated X apoptosis regulator (BAX); **f** tumor necrosis alpha (TNFA); **g** anti-Müllerian hormone (AMH); **h** BCL2 apoptosis regulator (BCL2); and **i** superoxide dismutase 1 (SOD1)

Among the four gene markers investigated for follicular development, ovarian function, and cell growth and development (*FSHR*, *LHCGR*, *AMH*, and *IGF1*), only *IGF1* exhibited a statistically significant lower expression in experimental subgroup 2 compared with both the control group [median (IQR); 0.19 (0.42) vs. − 1.67 (3.22); *p* = 0.003] and the cold ischemia group [median (IQR); 0.06 (0.39) vs. − 1.67 (3.22); *p* = 0.011]. This difference was not observed in subgroup 1 (Fig. 8b)*.*

Examining one apoptosis gene marker (*BAX*) and a proliferation marker (*BCL2*) revealed that *BAX* exhibited a significant upregulation in the ovaries from experimental subgroup 2 compared with ovaries exposed to the cold ischemia [median (IQR); 0.35 (0.16) vs. − 0.13 (0.2); *p* = 0.035] and compared with control ovaries [median (IQR); 0.35 (0.16) vs. − 0.13 (0.34); *p* = 0.016] (Fig. 8e). Meanwhile, the *BCL2* gene remained similar between the groups (Fig. 8h). The hypoxia marker *HIF1A* demonstrated a significant upregulation in the ovaries from experimental subgroup 1 compared with the non-perfused ovaries; control ovary [median (IQR); 1.63 (0.07) vs. 0.73 (0.4); *p* = 0.001] and ovary after cold ischemia [median (IQR); 1.63 (0.07) vs. 0.84 (0.26); *p* = 0.003] (Fig. 8c).

Our analyses revealed no variations in the expression of pro-inflammatory and antioxidant gene markers *TNFA* and *SOD1* (Fig. 8f, i).

## Discussion

### Ovary collection and preservation

The results of this study build upon our previous experiments [15], demonstrating that by extending ex vivo ovarian perfusion time and refining gonadotropin stimulation protocols, it is possible to obtain MII oocytes from ewe ovaries. A key methodological advancement in the present study is the introduction of an overnight pause in gonadotropin administration, which, for the first time, resulted in the successful retrieval of MII oocytes. Notably, these oocytes were obtained from an ovary that underwent a 5-day perfusion without continuous gonadotropin exposure. These findings indicate that a single daily dose of gonadotropins might be more effective in promoting oocyte maturation compared to continuous daily administration at the same concentration. However, due to the current limited sample size, these observations are insufficient to draw definitive conclusions. These refinements align with the physiological pattern of gonadotropin secretion in cycling ewes, which is characterized by daily peaks [16]. Building on these insights, the present study aimed to improve the stimulation protocol by implementing pulsatile gonadotropin administration, with the goal of enhancing follicular development and increasing the retrieval rate of mature oocytes (MII).

### Perfusion setup

Obtaining MII oocytes and a high number of oocytes after ex vivo perfusion of ewe’s ovaries for up to 7 days represents a significant milestone for improving ex vivo ovarian perfusion protocols and underscores the potential of this model to support advanced follicular development and successful oocyte maturation. The absence of an overnight stimulation of ovaries in subgroup 2 might have facilitated the enhanced follicular maturation and improved oocyte quality compared to ovaries in subgroup 1; however, further investigation is warranted. Notably, variations in sheep breeds present at the slaughterhouse, including Texel, Dorper, Dorset, and Swedish Landrace breeds, may contribute to some of the observed differences between the study samples since some breeds are specifically bred for a year-round breeding capability (e.g., Texel, Dorper, and Dorset), and may therefore exhibit more favorable outcomes in terms of oocyte retrieval and maturation due to their inherent reproductive characteristics. Conversely, Swedish Landrace sheep, characterized by a delayed estrous period compared to other breeds, may yield less favorable results in terms of oocyte retrieval and maturation. Therefore, further research examining the breed-specific reproductive physiology and response to ovarian stimulation protocols is essential to fully elucidate this potential influence on oocyte quality and maturation outcomes.

### Gene expression analysis

This study also incorporated gene expression analysis of apoptosis and hypoxia markers, offering deeper insights into ovarian health during perfusion compared to our previous study [15]. We aimed to explore the variation in gene expression within perfused ovaries compared to control and cold ischemia groups. Among the examined genes associated with follicular development and cell growth, *IGF1* exhibited lower expression levels in both experimental subgroups, with a significantly greater reduction observed in subgroup 2. Notably, the expression levels in subgroup 2 were markedly lower compared to both the fresh control and the cold ischemia control groups. Prior research has underscored the pivotal role of *IGF1* in promoting primordial follicle activation and cell proliferation [23]. The observed lower gene expression levels in our study suggest a potential disruption in follicular development due to the ex vivo perfusion conditions, potentially compounded by insufficient gonadotropin stimulation [24]. Thus, refinement of the protocol is still needed. This interpretation finds support in histomorphological findings indicating a reduced cell proliferation and an increased apoptosis in the immature follicular stages. Similarly, the apoptosis gene marker *BAX* exhibited a significant upregulation in experimental subgroup 2 compared to both the control and the ischemia group, while the anti-apoptotic *BCL2* gene expression remained unchanged. These results suggest a potential imbalance between pro- and anti-apoptotic pathways, leading to heightened apoptotic activity within the perfused ovaries of experimental subgroup 2, with potential implications for follicle survival [25, 26].

Moreover, there was a notable increase in the hypoxia marker *HIF1A* expression in experimental subgroup 1 compared to both control ovaries and ovaries exposed to ischemia. *HIF1A* has been linked to follicular growth [27], and the increase may also be a protective response to the hypoxic conditions induced by cold ischemia followed by the reperfusion events [28]. Our analyses did not reveal significant variations in the expression of endothelial cell-related pro-inflammatory and antioxidant gene markers *TNFA* and *SOD1* among the experimental subgroups. This suggests that the experimental conditions did not induce substantial changes in endothelial cell function or a significant oxidative stress within the ovarian tissue [29]. However, it is noteworthy that our analysis primarily focused on ovarian cortex tissue. The response of endothelial cell-related markers may vary between ovarian stromal and vascular tissue. Additionally, within the vascular tissue, different tissue types, such as macrovasculature (in the ovarian pedicle) and microvasculature (in the ovarian medulla), may exhibit distinct responses to perfusion [29].

### Histomorphological and vascular integrity

In terms of ovarian histomorphology, our findings indicate that the cold ischemia did not induce significant histomorphological damage compared to control tissue. This suggests that the ovaries can tolerate a cold ischemia time of at least 4 h without major histological changes following reperfusion. Furthermore, the perfusion was able to sustain the integrity of non-damaged primordial, primary, and antral follicles, supported by a comparable proliferation level (Ki-67 staining) to the control groups, which suggests a healthy follicular condition throughout the perfusion [30]. However, fewer non-damaged secondary follicles were observed in subgroup 2 compared to cold ischemia, perhaps due to the lack of follicular development in response to omitted overnight gonadotropin stimulation [31]. This was further supported by a decrease in ongoing cell proliferation, indicated by significantly lower Ki-67 staining. Despite this, subgroup 1, with overnight stimulation, exhibited some non-damaged secondary and antral follicle development, though not statistically significant. The increase in damaged G1 secondary follicles after perfusion in both subgroups, and G2 primordial follicles in subgroup 1, aligns with prior observations [15], suggesting increased vulnerability of these follicular classes to perfusion-related damage. This was further supported by the significantly higher cleaved caspase 3 staining observed in damaged secondary follicles of subgroup 2 compared to the control groups, indicating increased apoptotic activity. This suggests that overnight gonadotropin exposure, as in subgroup 1, may not fully ensure immature follicle preservation. Regarding vessel integrity, significant differences were observed among the subgroups, with subgroup 2 showing a markedly higher percentage of injured medullary arteries and veins compared to both the control ovary and the ovary subjected to cold ischemia, where no vascular injuries were detected. However, despite these differences in vessel injury within the medullary region, the histomorphology of ovarian arteries and veins was preserved across all experimental groups, suggesting that only medullary vessels are more vulnerable to injury under certain conditions. The preservation of vascular integrity is a notable outcome, especially given the extent of ischemic insult and prolonged perfusion durations studied. It highlights the effectiveness of the perfusion technique in maintaining the overall vascular architecture and supporting follicular survival, while reducing tissue damage, consistent with our previous findings [15].

### Hormonal dynamics

The assessment of steroid production during ovarian perfusion offers valuable insights into the functionality of ex vivo systems and their capacity to replicate in vivo hormone dynamics [21, 32–34]. In this investigation, analysis of the perfusion medium revealed consistent patterns in estrogen and progesterone concentrations within and across experimental subgroups throughout the perfusion periods. Our earlier study indicated stable or decreased estrogen levels during perfusion, likely attributed to insufficient gonadotropin follicle stimulation and repeated perfusate changes every 24 h, which may have diluted estrogen concentrations. Conversely, progesterone concentration increased during perfusion, indicating early luteinization processes [15]. However, in the current study, estrogen concentrations did not significantly rise during perfusion in either experimental subgroup, perhaps secondary to frequent perfusate changes. The absence of an early increase in progesterone levels in the perfusate was consistent with no signs of premature ovulation or luteinization before OPU. Frequent perfusate exchanges were implemented to prevent the accumulation of potentially toxic concentrations of lactate and other metabolic byproducts, given the high ovarian metabolic rate and lack of systemic clearance mechanisms in the ex vivo setting. Although lactate concentrations did not significantly differ within or between experimental subgroups, the more pronounced increase observed in subgroup 1, along with higher expression levels of the hypoxia-related gene *HIFA*, may indicate inadequate oxygen supply. However, it is notable that hormone concentrations in the perfusion medium were significantly lower compared to ewe plasma levels. This indicates potentially inadequate gonadotropin supplementation of the perfusate, with FSH and LH, to support sufficient follicle stimulation and steroid production. Thus, optimization of ovarian stimulation protocols with appropriate gonadotropin concentrations is imperative.

### Clinical implications

The strengths of this study include the achievement of mature oocyte retrieval in ex vivo ovarian perfusion, representing a significant advancement in the field. Additionally, the comprehensive evaluation of follicular integrity, oocyte maturity, and gene expression provides valuable insights into the mechanisms underlying ovarian function during perfusion. The limitations of our study include potential variations in outcomes due to differences in sheep breeds. Additionally, we exclusively used adult ewes; therefore, it would be valuable to extend this ex vivo perfusion model to pre-pubertal ovaries to assess its potential translational relevance for pre-pubertal girls. Moreover, further optimization of gonadotropin stimulation protocols is necessary. By optimizing the gonadotropin stimulation protocol for ex vivo preserved ewe ovaries, we achieved a significant milestone by successfully retrieving mature MII oocytes. These findings underscore the potential of ex vivo ovarian perfusion models to support advanced follicular development and oocyte maturation. Such models offer promising prospects for fertility preservation in cancer patients, providing a viable alternative to traditional approaches. Further research is needed to address protocol optimization and inter-individual variability, ensuring wider clinical applicability.

## Conclusion

This study highlights the feasibility of retrieving mature MII oocytes through ex vivo ovarian perfusion, marking a critical advancement in reproductive science. Our findings underscore the importance of pulsatile gonadotropin stimulation and breed-specific factors in enhancing follicular development and oocyte quality. The limitations of the study, including small sample sizes, breed-related variability, and suboptimal hormonal dynamics, point to areas requiring further investigation. Future research should aim to refine stimulation protocols, explore the role of genetic and breed-specific factors, and address the physiological challenges of ex vivo systems. With continued optimization, this approach holds great promise for developing innovative fertility preservation methods, especially for individuals facing gonadotoxic treatments.

## Supplementary Information

Below is the link to the electronic supplementary material.

Supplementary file1 (DOCX 16 KB)

## Data Availability

All data supporting the findings of this study are available within the paper and its Supplementary Information. Primer sequences are provided in Supplementary Table 1.

## Code availability

All data supporting the findings of this study are available within the paper and its Supplementary Information. Primer sequences are provided in Supplementary Table 1.
