# Supplementary material for: Ovarian function and response to gonadotropins after prolonged perfusion of whole ewe ovaries in a bioreactor
Source: J Assist Reprod Genet. 2025 Feb 26;42(5):1491–507. doi: 10.1007/s10815-025-03432-6 (PMC12167194; doi:10.1007/s10815-025-03432-6)
Supplement: Supplementary file 1 — Supplementary file1 (DOCX 16 KB) [file 10815_2025_3432_MOESM1_ESM.docx]

**Supplementary Table 1** Target genes and specific primer sequences. The target genes included follicle-stimulating hormone receptor (FSHR), luteinizing hormone/choriogonadotropin receptor (LHCGR), anti-Müllerian hormone (AMH), insulin-like growth factor 1 (IGF1), B cell lymphoma protein 2 (BCL2), BCL2-associated X protein (BAX), hypoxia-inducible factor 1 subunit alpha (HIFA), superoxide dismutase 1 (SOD1), and tumor necrosis factor alpha (TNFA). Peptidyl-prolyl cis-trans isomerase H (PPIH) was utilized as the reference gene. Fw: Forward Primer; Rv: Reverse Primer.

| **Target Gene** | **Primer Sequences 5' → 3'** |
| --- | --- |
| **FSHR** | Fw: TCT GTG GCT GCT ATA CTC  Rv: TTG GCT ATC TTG GTG TCA |
| **LHCGR** | Fw: TCC TCA TCT TCA CCG ATT  Rv: AGA GAA CCA GTA GAA CCT T |
| **AMH** | Fw: ACA TAC CAG GCC AAC AAC  Rv: ATC TTC AGC AGC AC |
| **IGF1** | Fw: CCA AGG CTC AGA AGG AAG  Rv: CTT CGC TCT TTA GGA AGG G |
| **BCL2** | Fw: CCC GCC ACG AAT TAC TAA G  Rv: CAC TCC AGC CAA CAG TAT G |
| **BAX** | Fw: ACG GTG ACC TTT GTG  Rv: AGT CCA AGG CAG TTG ATG |
| **HIF1A (formerly HIFA)** | Fw: GTC ACT TTG CCA GCT CA  Rv: GGG TGG GCA GAA CAT TTA |
| **SOD1** | Fw: AAG TCG TCG TAA CTG GAT  Rv: CAG CCT TGT GTA TTG TCT C |
| **TNFA** | Fw: GAT AAC CTC CCC AGA CAC  Rv: TCA GAA GGA TCC AAG TCC A |
| **PPIH (reference)** | Fw: ATT CAA GCC CTG TCA ATC C  Rv: CGT CTG CAA AGA GCT CAA |
